# Anti-mycotoxin additive mixture in cattle feed contaminated with multiple mycotoxins: impacts on performance and health

**DOI:** 10.1007/s11250-026-04921-1

**Published:** 2026-02-26

**Authors:** Mario Augusto Torteli, Andrei L. R. Brunetto, Emeline P. Mello, Guilherme Luiz Deolindo, Luisa Nora, Tainara Letícia dos Santos, Eduardo Micotti Da Gloria, Daiane Manica, Margarete Dulce Bagatini, Aleksandro Schafer Da Silva

**Affiliations:** 1 Programa de Pós-graduação em Zootecnia, Universidade do Estado de Santa Catarina (UDESC), Chapecó, SC Brazil; 2 Programa de Pós-graduação em Bioquímica e Biologia Molecular, UDESC, Lages, SC Brazil; 3 Department of Biological Science, Luiz de Queiroz” College of Agriculture, University of São Paulo, Piracicaba, São Paulo 13418-900 Brazil; 4 Postgraduate Program in Biochemistry, Federal University of Santa Catarina, Florianópolis, SC Brazil; 5 Centro de Ciências da Saúde, Universidade Federal Fronteira Sul, Chapecó, Brazil

**Keywords:** Cattle, Fungi, Animal production, Contaminants, Animal health

## Abstract

This study investigated the impact of including an anti-mycotoxin additive on the performance and health parameters of Holstein cattle in the rearing phase, fed diets contaminated with multiple mycotoxins. A diet contaminated with aflatoxins (200 ppb), fumonisins (15 ppm), zearalenone (500 ppb), deoxynivalenol (1.5 ppm), ochratoxin A (100 ppb), and T-2 toxin (300 ppb) was fed to growing cattle for 87 days. The experiment involved 24 cattle divided into three groups: negative control (CONT), positive control (MYCO: with mycotoxin blend), and test group (MYCO+ADDI: with mycotoxin blend + anti-mycotoxin additive). The additive’s formulation contained yeast cell wall, bentonite, modified montmorillonite, activated charcoal, milk thistle extract (*Silybum marianum*), and a selenium-amino acid complex. At strategic points, production performance data (weight gain, daily intake, and feed efficiency) and animal health biomarkers (hematology, clinical biochemistry, and oxidative stress markers) were assessed. Cattle in the MYCO group had lower average daily gain and feed efficiency, but the additive (MYCO+ADDI) minimized this negative impact of mycotoxins. Lower total leukocyte and lymphocyte counts were observed in cattle in both groups that consumed the mycotoxin-contaminated diet. Creatine kinase activity was higher in animals the MYCO and MYCO+ADDI groups compared to CONT. Alanine aminotransferase and gamma-glutamyl transferase activity was higher in the blood of cattle in the MYCO group compared to other groups. Aspartate aminotransferase activity was higher in MYCO group compared to CONT; but no difference between MYCO+ADDI. Levels of reactive oxygen species, lipid peroxidation, and myeloperoxidase activity were higher in the serum of cattle in the MYCO group compared to the other groups. Therefore, it can be concluded that the strategic use of an anti-mycotoxin additive minimized the damage caused by exposure to six concomitant mycotoxins, reducing liver damage and oxidative reactions.

**Supplementary Information:**

The online version contains supplementary material available at https://doi.org/10.1007/s11250-026-04921-1.

## Introduction

Ensuring the global food supply, balancing productivity, animal welfare, and sustainability, is one of the greatest challenges facing modern agriculture (FAO 2023). In Brazil, both milk and beef production have advanced in the pursuit of greater efficiency and quality. To achieve this goal, it is essential to invest in nutritional strategies that enhance animal performance. Among the main obstacles are mycotoxin poisoning, invisible contaminants that affect herd health, compromise livestock performance, and reduce the quality of the final product.

Mycotoxins are toxic substances produced by filamentous fungi such as *Fusarium*,* Aspergillus*,* Penicillium*, and *Alternaria*; notably aflatoxins, fumonisins, zearalenone, desoxynivalenol, ochratoxins, and trichothecenes type T-2. These toxins directly affect human and animal health, compromising food and agricultural productivity (Křížová et al. 2021). Aflatoxins (B1, B2, G1, and G2) produced by *Aspergillus* sp. are hepatotoxic and immunosuppressive, with deleterious effects on milk production and increased abortion rates in cows (Whitlow and Hagler 2008). Fumonisins, derived from *Fusarium* sp. can cause liver and enteric dysfunction and are associated with pulmonary edema and cardiomyopathies (Křížová et al. 2021). Trichothecenes (such as T-2 and HT-2) can cause hemorrhagic gastroenteritis, anorexia, and embryonic malformations, in addition to acting as potent inhibitors of protein synthesis (Křížová et al. 2021). Zearalenone (ZEA), due to its estrogenic action, interferes with the reproductive cycle of cattle, promoting abnormal estrus, abortions, and premature mammary development (Dallmann et al. 2021). Deoxynivalenol (DON), also derived from *Fusarium* sp., affects gastrointestinal mucosa, compromising nutrient absorption and altering ruminal fermentation; while ochratoxin A (OTA) mainly affects the kidneys, causing nephropathies and immunosuppression (Whitlow and Hagler 2008).

Therefore, the presence of these substances poses a risk not only to animal health and welfare but also to the food safety of human consumers. Strategies such as the use of mineral adsorbents, humidity control, and input monitoring are recommended to mitigate the effects of these toxins (Keller et al. 2021). Despite advances in research on fungal contamination control, studies evaluating, in a single experiment, the efficacy of an anti-mycotoxin additive against six different mycotoxins are still rare. Most available studies focus on isolated toxins or partial combinations, which limit their practical applicability in intensive animal production systems. This gap highlights the need for integrated studies that reflect the real challenges of the livestock environment.

The synergistic blend of additives aims to reduce the availability and toxicity of mycotoxins in the gastrointestinal tract and hepatic metabolism of animals, as was the focus of the anti-mycotoxin additive used in this study. The yeast cell wall, rich in β-glucans, can adsorb lipophilic mycotoxins through hydrophobic interactions and hydrogen bonds (Dall’erta et al., 2023). Modified bentonite or montmorillonite can sequester polar compounds through a strong cationic charge and interlayer sieving effect (Chen et al. 2021). Activated charcoal, with microporosity greater than 1,000 m²/g, ensures broad-spectrum adsorption without compromising nutrient absorption (Kihal et al. 2023). Milk thistle extract stimulates phase II enzymes and preserves glutathione stores, protecting the liver against oxidative stress and favoring the biotransformation of toxins (Sun et al. 2021). Finally, the selenium-amino acid complex is efficiently incorporated into glutathione peroxidases, strengthening the systemic antioxidant system and contributing to the indirect inactivation of mycotoxins (Pereira et al. 2024). Therefore, this study aimed to determine whether the inclusion of an anti-mycotoxin additive in contaminated diets can mitigate the adverse effects of these toxins, resulting in improvements in production performance and animal health indicators.

## Materials and methods

### Mycotoxin production

Aflatoxins were produced by an isolate of *Aspergillus nomius*, Fumonisins were produced by an isolate of *Fusarium verticilloides*, Desoxynivalenol and Zearalenone by an isolate of *Fusarium graminearum*, Ochratoxin A by an isolate of *Aspergillus ochraceus* and Toxin T-2 by an isolate of *Fusarium sporothricoides.* Fungal isolates were used to ferment converted rice for Aflatoxins, Ochratoxin A, Desoxynivalenol and Zearalenone production. For Fumonisins and Toxin T-2 production broken maize was used. The following protocol was used for production: erlenmeyer flasks of 500 mL volume were used to receive 100 g of rice or broken maize. At least 2 h before the sterilization 40 mL of distillated water was added to flask and mixed with rice or broken corn. The sterilization was performed at 121 C for 30 min (CS -75, Prismalab, Rio de Janeiro - RJ, Brazil) and then the flasks were left to lose temperature before inoculation. The rice or broken maize were inoculated with 2 mL of 10^8^ spore mL^− 1^ of spore suspension of each fungus. Colonies of each fungus were grown in Potato Dextrose Agar for ten days before the suspension to be prepared. The incubation was carried out during 21 days at controlled temperature (25 °C) on static conditions. After incubation, fermented material was dried in oven at 57 °C and ground in a mill with < 0.85 mm sieve.

### Experiment

Twenty-four Holstein cattle (males) with seven months old and 208.4 ± 5.6 kg, raised at UDESC since birth, were used. In the experiment, we divided the cattle into three homogeneous groups with four pens, and two animals per pen: a negative control group (CONT: without added mycotoxin and without anti-mycotoxin); a positive control group (MYCO: with added mycotoxin); and a test group (MYCO+ADDI: addition of mycotoxins and anti-mycotoxins). The anti-mycotoxin additive (Blink Mycolink^®^ P, Blink Bioscience Brasil LTDA) is composed of yeast cell wall of *Saccharomyces cerevisiae* (Beta-glucans: + 140 g/kg), bentonite, activated charcoal, milk thistle extract, and selenium amino acid complex.

### Animal feeding

The mycotoxins and contamination concentrations used in this experiment were: aflatoxins (200 ppb), fumonisins (15 ppm), zearalenon (500 ppb), deoxynivalenol (1.5 ppm), ochratoxin A (100 ppb), and T2 toxin (300 ppb). Mycotoxins were added to the concentrate during its production. The anti-mycotoxin additive was used at a dose of 2 kg per ton of concentrate. Knowing the daily intake, we calculated the daily intake of the additive per animal, i.e., 15.6 g. For the experiment, we used a diet formulated according to the nutritional requirements of the animals (BR-CORTE) (Valadares Filho et al., 2016), with the diet formulated (Table 1) so that the cattle had a weight gain of 1.5 kg per day.

**Table 1:** Ingredients and chemical composition of the basal diet (corn silage + concentrate) used in this experiment

Itens	Proportion (%)
Corn silage	44.3
Concentrate^1^	55.7
Total	100
**Chemical composition**	
Dry matter (DM)	51.5
Crude protein, DM	12.6
Neutral detergent fiber, DM	29.4
Starch, DM	40.8
Ether extract, DM	3.70
Total Digestible Nutrients	72.0

NOTE 1: The concentrate was formulated based on ground corn (59.3%), soybean meal (18.1%), wheat bran (13.3%), soybean hulls (7.27%), vitamin-mineral core – *Novo Bovigold* (1.77%) and white salt (0.26%)

### Weighing, blood collection, and blood count

In the experiment, we collected samples and weighed animals four times (days 1, 30, 60, and 87). Weighing was performed using a digital scale, with the animals fasting for 12 h, to monitor performance. Based on this data, we calculated weight gain and average daily gain (ADG). Daily dry matter intake (DMI) was measured by pen. Based on these data, we calculated feed efficiency: ADG/DMI (kg/kg).

Blood samples were collected via the coccygeal vein using tubes containing EDTA and no anticoagulant. Hemoglobin, erythrocyte count, total leukocyte count, hematocrit, and leukocyte differentiation were measured immediately upon arrival at the laboratory using a 3-part Equip vet 3000 automatic hematology analyzer.

### Serum biochemistry and oxidative biomarkers

The tubes without anticoagulant were centrifuged (1980 *× g* for 10 min) to separate the serum for biochemical analysis. The supernatant was transferred to 1.5 mL microtubes, labeled, and stored at -20 °C until analysis. Serum levels of albumin (ALB), total bilirubin (BT), creatine kinase (CK-NAC), creatinine (CRE), gamma-glutamyl transferase (GGT), total protein (PT), aspartate aminotransferase (AST or TGO), alanine aminotransferase (ALT or TGP), and urea nitrogen (URE) were analyzed using an automated analyzer (Zybio^®^ EXC 200) and commercial kits (Analisa^®^). Globulin levels were obtained through mathematical calculation (total protein - albumin).

Serum samples were used to measure TBARS, ROS, and myeloperoxidase (MPO). Lipid peroxidation was analyzed in serum by the amount of thiobarbituric acid-reactive substances (TBARS), using the method of Jentzsch et al. (1996). To determine reactive oxygen species (ROS), we followed the technique described by Ali et al. (1992). In the presence of H_2_O_2_ as an oxidizing agent, MPO catalyzes the oxidative coupling of phenol and 4-aminoantipyrine (AAP), yielding a colored product, quinoneimine, with a maximum absorbance of 492 nm (Suzuki et al. 1983). MPO activity was analyzed using a modified peroxidase system, with a mixture of 12 µL of serum sample with 148 µL of AAP in phenol solution (2.5 mM AAP; 20 mM phenol) and 17 µL of H_2_O_2_ solution (17 mM). After 30 min of incubation at 37 °C, the system was read spectrophotometrically. The results were expressed as µM quinoneimine per mg of protein produced in 30 min (µMq/mg/30 min).

### Mycotoxin quantification in diets

For mycotoxins analyses samples of feed were ground to < 0.85 mm and one gram of the ground material was transferred to 50 mL test tube. It was added 10 mL of ultrapure water and 10 mL of acetonitrile/acetic acid (CH3CN: CH3COOH) [99.5:0.5, v/v] and the test tube was placed in a mechanical shaker for 10 min. A mixture of 4 g of MgSO_4_ and 1 g of NaCl was added and the tube was vigorously hand-shaken for 10 s. The solution was them centrifuged for 15 min at 5.000 x g, at 25 ◦C and 2.5 mL of supernatant was transferred to capped glass test tube where 2.5 mL of hexane was added. The solution was shaken for 2 h and then centrifuged at 1.000 x g, at 20 ◦C for 1 min. From lower phase (acetonitrile) 1 mL was withdraw and dried with Nitrogen (N2) stream at 40 ◦C. The reconstitution was performed with 75 µL of methanol in ultrasonic bath for 10s and 10s in test tube mixer after adding 75 µL of ultrapure water. After centrifugation for 10 min at 14.000 x g 60 µL was withdraw and transferred to vial where 140 µL of ultrapure water was added. Ten microlitres were injected in chromatographic system.

Detection and quantification of mycotoxins were performed with high-performance liquid chromatography coupled with tandem mass-spectrometry (LC/MS/MS). Chromatographic separation was carried out using Acqulty UPLC System (Waters, Milford, Massachusetts, EUA) equipped with 100 × 2.1 mm, 1.7 μm Acquity UPLC BEH C18 column, (Waters, Milford, Massachusetts, EUA). The column was maintained at 40 ◦C and the injection volume was 10 µL. The mobile phase consisted of 0.1% formic acid in water(A), and 0.1% formic acid in acetonitrile (B). The acetonitrile (B) concentration was raised gradually from 10% to 90% within 12 min, brought back to the initial conditions at 0,1 min, and allowed to stabilize for 3 min. The mobile phase was delivered at a flow rate of 0.4 mL/min. The LC system was coupled with Xevo TQS tandem mass spectrometer (Waters, Milford, Massachusetts, EUA), equipped with a turbo-ion electrospray (ESI) ion source. The mass-spectrometer was operated in scheduled multiple reaction monitoring (MRM) in positive mode. The data acquisition of mass spectrometer are showed in Table S1. Mycotoxins quantification was carried out using matrix-matched calibration curves, using extracts of diets phases not contaminated. The final contamination level observed in the contaminated diets is presented in Table 2.

**Table 2:** Mycotoxin contamination in cattle experimental diets

Diets	AFB1	AFG1	Total aflatoxins	DON	FB1	ZEA	OTA	T-2
CONT	ND	ND	-	218.7	171	ND	ND	ND
MYCO	91.0	136.0	227.0	1727.7	14,670	453.0	106.0	322.0
MYCO+ADDI	75.0	120.0	195.0	1481.3	15,332	486.0	87.3	301.0

ND = Not-detected. Limit of quantification AFB1 and AFG1 = 5.0 ppb/ DON = 100 ppb/FB1 = 100 ppb/ZEA = 20 ppb/ OTA = 5 ppb/ T-2 100 ppb

### Statistical analysis

All data were analyzed using the SAS^®^ MIXED procedure (SAS Inst. Inc., Cary, NC, USA; version 9.4), with Satterthwaite approximation to determine the denominator degrees of freedom for the fixed-effects test. Body weight, weight gain, and feed efficiency were tested for treatment fixed effects using pen (containing two animals within the treatment) as random effects. Blood data were analyzed as repeated measures and were tested for treatment, day, and treatment × day fixed effects, using animal (treatment) as random effects. The d1 results were included as an independent covariate. The first-order autoregressive covariance structure was selected according to the Akaike least information criterion. Means were separated using the PDIFF method (Tukey’s test), and all results were reported as LSMEANS followed by SEM. Significance was defined as *P* ≤ 0.05.

## Results

During the 87-day experimental period, all cattle remained free from clinical signs of intoxication, but detailed analyses revealed important subclinical changes.

### Animal performance

Dietary mycotoxin intake (MYCO) caused a significant lower in body weight gain (*P* < 0.05) compared to the CONT and MYCO+ADDI groups (Table 3). This decrease cannot be attributed to dry matter intake, which remained similar between treatments (*P* > 0.05). Consequently, a worsening feed conversion rate was observed, demonstrating lower efficiency in converting feed into live weight gain in the mycotoxin-exposed group (MYCO) compared to the other two groups.

**Table 3:** Body weight, weight gain, and feed efficiency of cattle fed a diet containing Mycotoxins (MYCO) and an anti-mycotoxin additive (MYCO+ADDI)

Variable	MYCO	MYCO+ADDI	CONT	SEM	*p*-value
Body weight, kg					0.58
d1	209.5	208.1	207.8	5.41	
d30	242.5	242.6	243.8	5.38	
d60	285.3	288.2	290.6	5.39	
d87	320.3	326	329.6	5.32	
Weight gain, kg					
d1-30	32.9	34.5	36.0	0.94	0.321
d1-60	75.7 b	80.0ab	82.7a	1.05	0.041
d1-87	110.7 b	117.8a	121.8a	1.02	0.028
d30-87	77.8 b	83.3a	85.8a	0.99	0.021
Average daily gain, kg					
d1-30	1.09	1.15	1.2	0.02	0.213
d1-60	1.26b	1.33ab	1.37a	0.04	0.022
d1-87	1.27b	1.35a	1.40a	0.04	0.021
d30-87	1.36b	1.46a	1.50a	0.03	0.018
Daily intake, kg of DM					
d1-87	7.14	7.12	7.21	0.02	0.97
d30-87	7.79	7.82	7.89	0.03	0.96
Feed efficiency, kg/k					
d1-87	0.178 b	0.19 a	0.194 a	0.005	0.01
d30-87	0.175b	0.187a	0.190a	0.003	0.01

Note: *P* ≤ 0.05 indicates statistically significant differences, illustrated by different letters within the same row

There were differences between the groups in weight gain and average daily weight gain. Regarding weight gain, from day 1 to day 60, the negative control group showed greater weight gain compared to the positive control group, but statistically similar to the test group. When we evaluated weight gain from day 1 to day 87, we found a statistical difference between the groups, with the negative control and test groups having greater weight gain compared to the positive control group. Also, regarding average weight gain, between days 30 and 87 of the experiment, we observed that the negative control and test groups (anti-mycotoxin) were superior to the positive control group.

### Hematology

A significant interaction between treatment and collection day (*P* < 0.01) was identified for total leukocytes and lymphocytes, with lower cell number in cattle in the MYCO and MYCO+ADDI groups, indicating possible immunosuppression resulting from toxin ingestion (Table 4). In contrast, there was no significant treatment effect or interaction for granulocytes, monocytes, erythrocytes, hemoglobin, hematocrit, or platelets, suggesting that the hematologic response was selective for components of the immune system.

**Table 4:** Hemogram of cattle fed a diet containing Mycotoxins and an anti-mycotoxin additive

Variable	MYCO	MYCO+ADDI	CONT	SEM	*p*-treat	*p*-treat x day
Total leukocytes (×10³)					0.01	0.01
d1	23.0	22.9	23.6	2.15		
d30	12.3b	11.9b	17.7a	1.11		
d60	9.5b	10.1b	15.0a	0.89		
d87	7.55b	7.74b	9.02a	0.38		
Lymphocytes (×10³/µL)					0.01	0.01
d1	15.3	13.4	15.1	2.14		
d30	9.17b	8.51b	13.8a	0.83		
d60	5.28b	5.97b	8.36a	0.54		
d87	4.07b	4.55ab	4.94a	0.22		
Granulocytes (×10³/µL)	2.84b	2.57b	3.57a	0.22	0.05	0.23
Monocytes (×10³/µL)	0.93	0.93	1.39	0.74	0.42	0.29
Total erythrocytes(×10⁶/µL)	8.42	7.31	8.11	0.17	0.15	0.35
Hemoglobin (mg/dL)	12.1	10.4	11.7	0.25	0.11	0.31
Hematocrit (%)	33.3	28.5	32.4	0.96	0.11	0.16
Platelets (×10³/µL)	349.0	351.0	354.0	18.7	0.92	0.95

Note: *P* ≤ 0.05 indicates a statistically significant difference, illustrated by different letters within the same row

### Biochemistry

Serum parameters such as albumin, total protein, globulins, bilirubin, creatinine, and urea nitrogen did not change between treatments (*P* > 0.05). However, creatine kinase (CK) activity increased significantly on day 87 in the MYCO and MYCO+ADDI groups (*P* < 0.05). The liver enzymes aspartate aminotransferase (AST) and gamma-glutamyltransferase (GGT) also showed a treatment effect and interaction with time; AST activity was higher in the MYCO group on days 60 and 87 (*P* < 0.05) compared to the CONT group, while GGT was higher on days 30 and 60 in the MYCO group compared to the other groups. ALT had a treatment effect, greater activity in the blood of cattle in the MYCO group compared to the other groups (Table 5).

**Table 5:** Clinical biochemistry of cattle fed a diet containing Mycotoxins and an anti-mycotoxin additive

Variable	MYCO	MYCO+ADDI	CONT	SEM	*p*-treat	*p*-treat x day
Albumin (g/dL)	2.83	2.9	2.79	0.022	0.81	0.58
Total protein (g/dL)	6.08	6.12	5.92	0.051	0.86	0.72
Globulin (g/dL)	3.25	3.22	3.13	0.056	0.91	0.89
Bilirubin	0.04	0.04	0.03	0.001	0.97	0.98
Creatinine (mg/dL)	0.71	079	0.73	0.014	0.93	0.96
Urea (mg/dL)	11.3	11.7	12.4	0.409	0.95	0.81
ALT (U/L)	27.1^a^	23.1^b^	24.3^b^	0.415	0.05	0.18
Creatine kinase (U/L)					0.45	0.01
d1	394.0	415.0	383.0	29.6		
d30	245.0	277.0	254.0	18.4		
d60	229.0	234.0	220.0	9.47		
d87	307.0a	292.0a	218.0b	12.9		
AST (U/L)					0.01	0.01
d1	85.0	82.4	82.6	2.43		
d30	85.4	87.3	80.3	2.21		
d60	94.3a	91.1ab	88.3b	1.32		
d87	110.0a	99.9ab	90.4b	3.43		
GGT (U/L)					0.01	0.01
d1	16.2	15.8	13.6	0.901		
d30	19.7a	21.5a	13.7b	1.33		
d60	26.0a	24.3a	13.1b	1.92		
d87	24.5a	16.1b	14.9b	1.32		

Note: *P* ≤ 0.05 indicates statistically significant differences, represented by different letters within the same row

### Oxidative stress

There was a treatment × day interaction (*P* < 0.05) for reactive oxygen species (ROS), TBARS, and myeloperoxidase (MPO) activity (Table 5). On day 87, cattle in the mycotoxin group exhibited marked elevations in ROS, TBARS, and MPO compared to the other groups, demonstrating systemic oxidative stress even in the absence of apparent clinical signs (Table 6).

**Table 6:** Oxidative status of cattle fed a diet containing Mycotoxins and an anti-mycotoxin

Variable	MYCO	MYCO+ADDI	CONT	SEM	*p*-treat	*p*-treat x day
ROS (fluorescence)					0.53	0.01
d1	13.4	13.1	12.5	0.74		
d30	9.87	9.45	10.7	0.72		
d60	10.4	10.4	10.6	0.69		
d87	11.8a	8.39b	8.42b	0.66		
TBARS (nmol/mL)					0.47	0.01
d1	9.72	10.4	9.63	2.56		
d30	10.7	9.48	10.4	2.39		
d60	8.26	8.73	8.41	2.52		
d87	249a	16.1ab	10.4b	4.12		
Myeloperoxidase (µM of quinoneimine/30 min)					0.55	0.01
d1	3.23	2.72	2.36	0.35		
d30	4.01	2.55	3.25	0.35		
d60	1.93	1.78	1.96	0.33		
d87	4.33a	2.49ab	1.87b	0.41		

Note: *P* ≤ 0.05 indicates statistically significant differences, represented by different letters within the same row

### Practical implications

Chronic exposure to mycotoxins has induced interconnected subclinical effects that compromise cattle health and productivity. A decrease in weight gain and worsening feed conversion were observed, accompanied by markers of immunosuppression, liver and muscle cell damage, and increased systemic oxidative stress.

## Discussion

The decrease in weight gain and average daily gain in the positive control group can be explained by a set of systemic effects, primarily due to the action of the mycotoxins deoxyvalenol and zearalenone. These toxins bind to the ribosomes of intestinal cells, inhibiting protein synthesis and impairing epithelial renewal. As a result, the nutrient absorption surface is reduced, compromising the digestion and absorption of amino acids, carbohydrates, and lipids (Gallo et al. 2022). Ruminal microbiota becomes imbalanced, reducing dry matter digestibility, volatile fatty acid (VFA) production, and microbial protein synthesis (Assis et al. 2019).

According to Gallo et al. (2022), there is an increase in intestinal permeability, which stimulates local inflammation. This inflammatory process consumes energy that, in healthy animals, would be allocated to growth and the deposition of muscle mass and fat. Furthermore, when mycotoxins reach the liver, they activate biotransformation enzymes and generate reactive oxygen species, generating oxidative stress, forcing the body to release more energy in detoxification and cellular repair processes. The animals’ dry matter intake showed no statistical difference between treatments throughout the experimental period, thus not explaining the reduced weight gain in the positive control group compared to the other treatments. Unlike the study by Vedovatto et al. (2020), which states that the reduced weight gain is explained by the reduced dry matter intake in animals that ingest feed contaminated with mycotoxins, prolonged ingestion of these contaminants can cause a chronic or acute infection, depending on the animal’s immune system. Since the reduction in weight gain was not explained by the decrease in dry matter consumption, one of the points that may explain this reduction is the impact of mycotoxins on food digestibility, as mentioned above.

According to Riccio et al. (2014) and Zhang et al. (2023), mycotoxins compromise rumen cellulolytic bacteria, reducing the digestibility of neutral detergent fiber (NDF) and acid detergent fiber (ADF). Pinotti et al. (2014) demonstrated in their study that silymarin exerts potent hepatoprotective action by preserving liver function in toxin metabolism. In our experiment, this effect was confirmed by the reduction in ALT, AST and GGT, indicating greater liver efficiency. By maintaining hepatic metabolism at full capacity, silymarin attenuated mycotoxin-induced changes in the rumen microbiota, resulting in weight gain and feed efficiency similar to those of the negative control group.

Exposure to mycotoxins (MYCO and MYCO+ADDI) led to a marked reduction in total leukocyte counts, especially lymphocytes, in the early stages of the experiment. However, by day 87, the lymphocyte counts of cattle that consumed the additive were similar to those of the control group. This pattern is consistent with a transient immunosuppressive effect resulting from the action of mycotoxins on lymphoid organs such as the thymus, spleen, and lymph nodes, followed by compensatory mechanisms of functional reorganization of these tissues. Recent studies in ruminants reinforce this dynamic of initial lymphopenia and late recovery. Ren et al. (2022) described a 35% decrease in CD4 + T and B lymphocyte proliferation in Holstein dairy cows exposed to aflatoxin B1 by week 4, with a return to baseline levels after week 12. Zhang et al. (2023) reported a 22% decrease in circulating B lymphocytes in lambs chronically exposed to ochratoxin A until the 6th week, with complete recovery on the 90th day of the experiment. Silva et al. (2024) maintained goats under a low dose of aflatoxin B1 (50 µg/kg of feed) and documented initial suppression of CD4 + and CD8 + T lymphocytes during the first month, returning to the control level by the 87th day.

Exposure to mycotoxins can increase lipid peroxidation product (MDA) and inhibit antioxidant mechanisms, generating redox imbalance and oxidative stress (Mavrommatis et al. 2021). On day 87, an increase in ROS levels was observed in the mycotoxin group compared to the negative control. The group with the anti-mycotoxin additive showed even higher values, which may indicate a compensatory activation of the antioxidant pathway, as suggested by Gallo et al. (2022), who observed a transient intensification of oxidative activity in dairy cattle fed with plant extracts when faced with DON contamination. The consumption of the additive minimized the negative effects of mycotoxins, with ROS, TBARS, and MPO having intermediate values when compared to the other two groups, as the mycotoxin group had the highest values, reflecting greater oxidative damage, while the control group had lower values. A similar result was reported by Huang et al. (2023), who identified an increase in TBARS levels and a reduction in hepatic antioxidant activity in beef cattle exposed to aflatoxin B1. On the other hand, Zhang et al. (2024) observed a significant improvement in lipid peroxidation in lambs fed yeast cell walls when exposed to ochratoxin A, highlighting that the composition of the additive can modulate the magnitude of the antioxidant response. The mycotoxin group presented the highest MPO values, indicating systemic inflammation associated with neutrophil activation. The anti-mycotoxin group, in turn, presented values statistically similar to the control group, evidencing the protective effect of the additive in attenuating the inflammatory response. These findings are in line with the results of Haladi et al. (2023), who demonstrated a significant reduction in MPO and somatic cells in dairy cows fed antioxidant adsorbents in DON-contaminated diets. The significant interaction between time and treatments suggests that the effects of the protocols were not static throughout the experiment but varied according to the days evaluated. This temporal behavior is consistent with the data described by Custódio et al. (2020), who identified fluctuations in hematological and inflammatory parameters in Nellore cattle during 97 days of confinement, with progressive improvement correlated with the use of adsorbents with combined action.

The clinical signs of mycotoxins in cattle diets may go unnoticed, but their impact on productivity is measurable. Over the 87-day evaluation period, animals in the mycotoxin-exposed group lost an average of 11.1 kg each. Those receiving the anti-mycotoxin additive experienced a smaller loss of only 4.0 kg.

## Conclusion

The dietary supply of mycotoxins to the experimental group resulted in significant impairment of production parameters, characterized by a reduction in average daily gain and an increase in plasma markers of oxidative stress, indicating disturbances in redox homeostasis and potential cellular damage. In contrast, the group receiving the anti-mycotoxin additive showed statistically equivalent zootechnical performance to the negative control group, even under exposure to the toxins, demonstrating effectiveness in mitigating the deleterious effects. The reduction in the activity of liver extravasation enzymes reinforces the additive’s hepatoprotective action, possibly associated with the modulation of metabolic pathways involved in the inflammatory response and toxic biotransformation. These results highlight the potential of anti-mycotoxin additives as strategic nutritional tools to mitigate the impacts of subclinical mycotoxin poisoning, promoting the maintenance of liver function and productive efficiency in ruminant farming systems.

## Supplementary Information

Below is the link to the electronic supplementary material.


Supplementary Material 1


## Data Availability

The data are with the authors and can be available upon request.
