# Supplementary material for: Anti-mycotoxin additive mixture in cattle feed contaminated with multiple mycotoxins: impacts on performance and health
Source: Trop Anim Health Prod. 2026 Feb 26;58(2):142. doi: 10.1007/s11250-026-04921-1 (PMC12945937; doi:10.1007/s11250-026-04921-1)
Supplement: Supplementary file 1 — Supplementary Material 1 [file 11250_2026_4921_MOESM1_ESM.docx]

Table S1. Acquisition parameters data from mass spectrometer.

| **Analyte** | **MRM Transiction** | **Dwell Time (s)** | **Cone**  **Voltage (V)** | **Collision Energy (eV)** |
| --- | --- | --- | --- | --- |
| Aflatoxin B1 | 313.08>241.23 | 0.005 | 30 | 37 |
|  | 313.08>285.39 |  |  | 23 |
| IS Aflatoxin B1 | 330.00>300.90 | 0.005 | 30 | 23 |
| Aflatoxin B2 | 315.10>259.05 | 0.005 | 30 | 28 |
|  | 315.10>287.16 |  |  | 25 |
| IS Aflatoxin B2 | 332.00>303.10 | 0.005 | 30 | 25 |
| Aflatoxin G1 | 329.09>243.10 | 0.005 | 25 | 26 |
|  | 329.09>283.00 |  |  | 26 |
| IS Aflatoxin G1 | 346.00>257.00 | 0.005 | 25 | 26 |
| Aflatoxin G2 | 331.05>245.05 | 0.005 | 25 | 30 |
|  | 331.05>257.04 |  |  | 25 |
| IS Aflatoxin G2 | 348.00>330.00 | 0.005 | 25 | 25 |
| Ocratoxin A | 404.20>221.10 | 0.005 | 25 | 30 |
|  | 404.20>339.10 |  |  | 30 |
| IS Ocratoxin A | 424.00>250.00 | 0.005 | 25 | 25 |
| Deoxynivalenol | 297.19>231.18 | 0.037 | 20 | 10 |
|  | 297.19>249.18 |  |  | 10 |
| IS Deoxynivalenol | 321.10>263.00 | 0.039 | 15 | 10 |
| Zearalenone | 319.20>185.20 | 0.005 | 20 | 19 |
|  | 319.20>187.20 |  |  | 23 |
| IS Zearalenone | 337.00>199.10 | 0.005 | 20 | 19 |

| Fumonisin B1 | 722.78>334.29 | 0.005 | 30 | 40 |
| --- | --- | --- | --- | --- |
|  | 722.78>352.22 |  |  | 35 |
| IS Fumonisin B1 | 756.10>374.20 | 0.005 | 30 | 40 |
| Fumonisin B2 | 706.57>318.22 | 0.005 | 30 | 40 |
|  | 706.57>336.04 |  |  | 40 |
| IS Fumonisin B2 | 740.20>358.20 | 0.005 | 30 | 36 |
| Toxin T2 | 484.40>185.10 | 0.005 | 10 | 22 |
|  | 484.40>215.10 |  |  | 22 |
| IS Toxina T2 | 508.00>198.10 | 0.005 | 25 | 22 |
